# Creatine for the Treatment of Depression

**DOI:** 10.3390/biom9090406

**Published:** 2019-08-23

**Authors:** Brent M. Kious, Douglas G. Kondo, Perry F. Renshaw

**Affiliations:** 1 Diagnostic Neuroimaging, Department of Psychiatry, University of Utah, 383 Colorow Drive, Salt Lake City, UT 84108, USA; 2 George E. Wahlen Veterans Affairs Medical Center, 500 Foothill Drive, Salt Lake City, UT 84148, USA

**Keywords:** major depressive disorder, bipolar disorder, creatine, phosphocreatine

## Abstract

Depressed mood, which can occur in the context of major depressive disorder, bipolar disorder, and other conditions, represents a serious threat to public health and wellness. Conventional treatments are not effective for a significant proportion of patients and interventions that are often beneficial for treatment-refractory depression are not widely available. There is, therefore, an immense need to identify novel antidepressant strategies, particularly strategies that target physiological pathways that are distinct from those addressed by conventional treatments. There is growing evidence from human neuroimaging, genetics, epidemiology, and animal studies that disruptions in brain energy production, storage, and utilization are implicated in the development and maintenance of depression. Creatine, a widely available nutritional supplement, has the potential to improve these disruptions in some patients, and early clinical trials indicate that it may have efficacy as an antidepressant agent.

## 1. Introduction

Major depressive disorder (MDD) and associated syndromes, such as dysthymic disorder and bipolar depression, impact a substantial fraction of children and adults globally. Despite the widespread availability and utilization of conventional antidepressants such as the selective serotonin reuptake inhibitors (SSRIs) and serotonin-norepinephrine reuptake inhibitors (SNRIs), roughly 53% of persons with depression fail to respond to an initial trial of those medications [1], and as much as 35% of patients do not respond to multiple trials of different antidepressants [2]. Therapies that may be beneficial for treatment-refractory depression, such as electroconvulsive therapy or ketamine, are not widely available [3]. Most conventional antidepressants alter the release or reuptake of the monoamine neurotransmitters serotonin, norepinephrine, and dopamine. The limited efficacy of conventional antidepressants and the limited availability of more novel treatments with different mechanisms together demonstrate a crucial need to identify antidepressant interventions with different mechanisms of action which also have the potential to be accessible to many patients.

Although recently much work in the pharmacological treatment of depression has been devoted to studying the potential of medications that alter the activity of the glutamatergic system—especially, the anesthetic agent ketamine and its enantiomer esketamine, which are potent antagonists of the N-methyl-D-aspartic acid (NMDA) glutamatergic receptor [4,5]—other physiologic pathways may also contribute to the development of depression. In particular, as we review below, there is growing evidence that both unipolar and bipolar depression involve alterations in the regulation of brain energy stores, which could produce depression, or limit antidepressant response, by several routes. As a result of this research, a number of investigators have begun to examine the antidepressant potential of compounds that could improve brain bioenergetics—that is, the processes of brain energy storage, transport, and utilization. In particular, there has been increasing interest in the possible antidepressant efficacy of creatine (N-aminoiminomethyl-N-methylglycine). In what follows, we review the evidence that altered brain bioenergetics contribute to the pathogenesis of some cases of depression and to limitations on response to conventional antidepressants, examine the pharmacological properties of creatine relevant to its use as an antidepressant agent, examine pre-clinical evidence from animal studies and studies of healthy (non-depressed) humans that pertain to its potential antidepressant efficacy, and consider the limited but growing cadre of clinical studies that have examined creatine for the treatment of depression. This topic has previously been reviewed elsewhere [6,7]; the current review adds to these excellent papers in that it encompasses more information about neuroimaging findings in depression that indicate the presence of bioenergetics deficits, considers additional background pertaining to the role of hypoxia in the production of depression, and examines the results of the relevant clinical studies in more detail.

## 2. Methods

For this review, we identified empirical studies published in peer-reviewed journals in English using several search engines (PubMed, PsycINFO, and Google Scholar) encompassing publication dates up to June 30, 2019. The following initial search parameters were used: *depression creatine* OR *major depressive disorder creatine* OR *bipolar creatine* OR *suicide creatine*. The initial search revealed 733 records which were manually screened for relevance and duplication by the first author, leaving 112 records. Additional studies of relevance were identified through review of the reference lists of the studies identified in the initial search.

### 2.1. Creatine Biochemistry as it Pertains to Depression

Although the basic biochemistry of creatine is reviewed elsewhere in this issue (CITE), we will highlight a few facts that are pertinent to its possible role in the treatment of depression. Creatine is an organic acid that is synthesized from the amino acids arginine, glycine, and methionine. It is also derived from diet, particularly from foods containing meat and fish [8]. It is synthesized in brain to a limited degree, but brain levels are primarily maintained by active transport from the serum via the sodium- and chloride-dependent creatine transporter SLC6A8 [9,10]. The brain is an energetically demanding organ and accounts for approximately 20% of body energy consumption at rest, even though it accounts for roughly 2% of body mass [11,12]. The best-characterized role of creatine in energy metabolism is as an energy buffer: creatine is converted to phosphocreatine (PCr) by the creatine kinase reaction, thereby storing energy in a more stable form than is provided by adenosine triphosphate (ATP) [13]. The creatine kinase reaction occurs in muscles as well as in the brain [13]. Creatine also serves as an “energy shuttle,” as its more rapid rate of intracellular diffusion compared to ATP means that it is able to more efficiently transport energy from sites where it is synthesized (e.g., mitochondria) to sites where it is utilized (e.g., the neuronal membrane) along a concentration gradient [8,14]. Creatine kinase is most extensively expressed in brain regions that exhibit higher levels of activity, such as the hippocampus and cerebellum [15]. 

### 2.2. Animal Studies of Depression-Like Behavior

Animal studies clearly indicate the essential role of creatine, phosphocreatine, and the creatine kinase system in the regulation of behavior and in brain development [16,17], and have provided compelling evidence of the antidepressant effect of creatine. Allen et al. [18] evaluated the effect of creatine supplementation on depression-like behavior, measured via the forced swim test (FST), in rats. In their studies, the wire suspension test (WST) was used to control for motor ability. In one experiment, 30 female rats were given either no creatine, 2% creatine by weight, or 4% creatine by weight. In another experiment, 36 male rats were exposed to the same dietary protocols and behavioral tests. Female rats receiving 4% creatine exhibited significantly longer latency to immobility on the FST than controls, suggesting reduced depression-like behavior, though there was no difference between groups in the WST. Surprisingly, male rats maintained on 4% creatine showed reduced time to immobility and increased immobility in the FST, and again no difference in the WST. In a later study, the investigators used a similar protocol to assess the impact of creatine supplementation on response to the antidepressant fluoxetine. They found that female rats maintained on 4% creatine by weight for 5 weeks exhibited reduction in depressive behavior on the FST, and that the addition of creatine to fluoxetine enhanced the antidepressant effect of fluoxetine. Analysis of estrous cycle data for the animals indicated that ovarian hormones likely affected the response to creatine, with the antidepressant effects in females occurring in the proestrous and estrous phases [19]. To further explore the effect of gonadal hormones on creatine’s antidepressant efficacy, Allen and colleagues later conducted two related experiments. In the first experiment, male rats underwent either gonadectomy or sham surgery. Gonadectomized rats were then implanted with a supplemental testosterone capsule or an empty capsule. Sham-treated males did not demonstrate any significant change in performance on the FST with creatine supplementation, while gonadectomized males who received testosterone exhibited a non-significant trend toward reduced depressive behavior on the FST with increasing doses of creatine, and gonadectomized males who did not receive testosterone exhibited a non-significant trend toward worsening depressive behavior on the FST with increasing creatine doses. In the second experiment, female rats were either ovariectomized or sham-treated; a subset of those who were ovariectomized were treated with either estradiol, progesterone, the combination, or sesame oil vehicle only. Ovariectomized rats who received estradiol exhibited significantly fewer depressive symptoms than ovariectomized rats who received vehicle only. The investigators also found that creatine at both the 2% and 4% by weight doses reduced depressive behaviors in the ovariectomized rats who received estradiol and progesterone compared to no creatine; this effect was not observed in the other groups [20].

These studies of the antidepressant effects of creatine in animal models are supported by others that indicate that creatine and phosphocreatine levels are altered in animal models of depression. Using proton magnetic resonance spectroscopy (^1^H MRS), Kim et al. showed that mice exposed to the forced swim test exhibited reduced total creatine (creatine + phosphocreatine) levels in the left dorsolateral prefrontal cortex; this reduction was corrected by treatment with the tricyclic antidepressant desipramine [21]. Other studies of animal models of depression and chronic stress also tend to show reduced brain creatine concentrations. Rats exposed acutely to the forced swim test plus restraint stress and ether exhibit reduced creatine in the frontal cortex [22], including in the medial prefrontal cortex as measured by ^1^H MRS [23]. A social isolation model of depression, in which rats are reared in isolation from conspecifics for 8 weeks, showed that they exhibited reduced hippocampal (though not cortical) phosphocreatine, along with glutamate and glutamine, coupled with reductions in antioxidant enzymes and increases in brain levels of hydrogen peroxide [24]. Male tree shrews subjected to chronic social defeat stress (another model of depression) exhibited on average a 15% reduction in cerebral total creatine levels, which was associated with reductions in neurogenesis measured by immunohistochemistry for BrdUrd. These changes were prevented by treatment with tianeptine, a tricyclic antidepressant [25]. Other, similarly-designed studies using the social defeat stress model in tree shrews have also found reductions in cerebral creatine, which are attenuated by a variety of potential antidepressant compounds [26,27,28,29].

A large series of animal studies conducted by Rodriques and collaborators and designed to ascertain the mechanisms underpinning creatine’s antidepressant activity for rats in the tail suspension test (TST) also indicate that creatine has an antidepressant effect. These studies suggest that creatine may, in addition to its role in energy storage, function as a neurotransmitter. Almeida et al. [30] found that radiolabeled creatine is released from stimulated brain tissue in a fashion that appeared consistent with action-potential dependence—for instance, creatine was not released if the culture medium lacked calcium, which is necessary for the degranulation of synaptic vesicles. It has also been found that the antidepressant-like effect of exogenous creatine in mice in the TST is blocked by compounds that inhibit PKA, PKC, CAMK-II, and MEK1/2, a group of protein kinases that have been implicated in depression, suggesting that the antidepressant effect of creatine is mediated by these pathways [31]. The group later showed that the antidepressant effect of creatine in the TST was blocked by compounds that inhibit PI3K, such as wortmannin and rapamycin, indicating that its antidepressant effect involves Akt, mTOR, and GSK3, among other intracellular signals [32]. In a related study, they found that the effect of creatine in the TST in rats exposed to corticosterone was similar to that of ketamine (a novel antidepressant), and that these effects were reduced for both compounds by substances that targeted the PI3K/Akt and mTOR pathways [33,34]. These intracellular signals have been implicated in the pathogenesis of depression [35,36], potentially because of their effects on synaptic sprouting, mediated by BDNF [37].

Creatine may interact with other neurotransmitter systems, such as the monoamines and adenosine. The antidepressant effect of creatine in the TST is blocked by compounds that inhibit serotonin synthesis [38], and enhanced by co-administration with SSRIs like fluoxetine [38]. Likewise, haloperidol and other dopamine receptor antagonists reduce the anti-immobility effect of creatine in the TST, while this effect is enhanced by co-administration with dopaminergic compounds such as bupropion [39], implying that creatine may interact with the dopaminergic system. The antidepressant effect of creatine in a rodent model is also attenuated by compounds that block adenosine receptors, and enhanced by compounds that agonize those receptors [40,41]; adenosine receptors, too, have been implicated in the etiology of depression [42].

Finally, creatine may contribute to an antidepressant response by reducing oxidative and nitrosative stress. It has been observed that creatine can reduce glutamate-induced neuronal excitotoxicity, as it reduced the production of reactive oxygen species and mono-nitrogen oxides; this ability appeared to be dependent on its antioxidant effect, as it also reduced the effects of exposure to hydrogen peroxide [43].

### 2.3. Altered Brain Bioenergetics in Human Depression

Multiple sources of evidence, from epidemiology, genetics, biochemistry, and neuroimaging, indicate that bioenergetic abnormalities contribute to the development of depressive symptoms in both MDD and bipolar disorder (BD). Together, they suggest that compounds that might enhance brain energy storage, like creatine, could contribute to the treatment of depression.

Many clinical conditions that are associated with impaired energy storage and bioenergetics synthesis are also associated with depression. Depression frequently afflicts persons suffering from chronic medical illness, including both type 1 and type 2 diabetes [44,45]. Depression is three times more prevalent in people with type 1 diabetes than in the general population, and twice as common in type 2 diabetes [46]. Similarly, type 1 diabetes is associated with changes in energy metabolism, and mitochondrial function plays a primary role in the treatment and prevention of long term consequences of the disorder [47]. A case-control study showed that brain energy metabolism is abnormal in type 1 diabetes as represented by the PCr/ATP ratio [48]. Furthermore, phosphorus-31 magnetic resonance spectroscopy (^31^P MRS) studies in the heart showed altered energy homeostasis and decreased PCr/ATP ratios in type 1 diabetes patients, identical to the pattern observed in the brain [49,50,51].

Dietary patterns that may reduce creatine intake are also associated with the risk of depression in some studies, although results are mixed. Li and colleagues [52] observed that elderly men who followed a vegetarian diet had a higher risk of depression, more severe symptoms of depression, and increased scores on the Geriatric Depression Scale, though a similar result was not found for women. Matta et al. [53] also found evidence that a vegetarian diet was associated with increased depressive symptoms on the CES-D in a large (*n* = 90380) cross-sectional study of French persons, but observed, in addition, that any dietary restrictions were associated with increased symptoms of depression, not merely restriction of the intake of meat, fish, eggs, or dairy. Larssen and colleagues [54] reported that among 2041 Swedish and Norwegian students, vegetarian diet was associated with increased self-reported frequency of depressive episodes in both males and females. Although the apparent associations between depression and vegetarian or vegan diets may be related to nutrient deficiencies, including deficiencies of creatine intake, it has also been suggested that the onset of mental disorders may precede the adoption of a vegetarian diet in some cases [55].

Hibbeln and colleagues [56] found, in a sample of 9668 male participants in the Avon Longitudinal Study of Parents and Children, that persons with vegetarian diets (*n* = 350) had greater depression scores (on the Edinburgh Postnatal Depression Scale or EPDRS) and a greater risk of clinically significant depressive symptoms (EPDRS total score >= 10) than those with omnivorous diets, after adjusting for sociodemographic confounds. In a related study involving both men and women [57], however, there was no association between a vegetarian diet and developing clinically significant depressive symptoms on the EPDRS, defined in this case as scores > 12. Similarly, Jin et al. [58] noted that vegetarians in a sample of 892 participants in the Mediators of Atherosclerosis in South Asians Living in America (MASALA) study had 43% lower odds of exhibiting significant depressive symptoms, while Beezhold et al. observed, in a study of 138 Seventh Day Adventists [59], that vegetarians reported less negative symptoms than omnivores on both the Profile of Mood States (POMS) and the Depression Anxiety Stress Scale (DASS). In a related study, Beezhold and colleagues [60] conducted an online survey of persons who participated in diet-related social media sites and found that those who followed vegan and vegetarian diets had less anxiety and stress on the DASS than omnivores. Sanchez-Villegas and colleagues [61] reported that in the Seguimiento Universidad de Navarra (SUN) cohort study, adherence to a pro-vegetarian dietary pattern was associated with a reduced incidence of depression in 15,093 Spanish persons followed for an average of 8.5 years, while Velten et al. [62] found that having a non-vegetarian diet was associated with greater positive mental health in a sample of 15,396 German and Chinese students. Still, there is evidence that creatine supplementation can improve cognitive performance in vegetarians, as supplementation with creatine at 5 gm per day for 6 weeks improved performance on backward digit span and Raven’s Progressive matrices compared to placebo in vegetarian subjects [63].

Medical conditions associated with relative hypoxia, such as asthma and chronic obstructive pulmonary disease (COPD), are associated with increased rates of depression and suicide [64,65,66,67,68]. COPD is also linked to increased odds of suicidal ideation and suicide attempts compared to non-hypoxic chronic medical conditions [66,67], and the risk of depression in COPD is almost twice that in non-hypoxic illnesses [68]. Cigarette smoking, which causes relative hypoxia independent of associated lung diseases [69], is also linked to increased risk for suicide and depression [70]. In adolescents, smoking increases the odds of developing depression by 1.7 times compared to non-smokers [71]. Current smoking in adults is linked in a dose-dependent fashion to increases in suicide rates [72]; long-term abstinence reduces this risk, while relapse precedes a return to high risk [73]. Poorly-controlled asthma is also associated with an increased incidence of suicide rates compared to remitted asthma [64], and suicide rates for teens with asthma are more than double those of teens without asthma [65].

There is also extensive, and growing, evidence accumulated by our group and others that increased altitude of residence, which may be associated with chronic relative hypoxia, is a risk factor for depression, suicide, and related adverse psychiatric outcomes [74,75,76,77,78,79,80,81,82,83,84,85,86,87,88]. In conjunction with this, it has been show that simulated high altitude can produce depression in a rodent model, and that these symptoms are not responsive to most antidepressants [89,90,91,92]. ^31^P MRS studies have also indicated that increased altitude of residence is associated with alterations in cerebral bioenergetic signatures that are similar to those seen in depression (described below) [93,94].

There is increasing reason to believe that mood disorders themselves involve alterations in brain bioenergetics, in some cases related to underlying abnormalities in mitochondria and mitochondrial activity, including changes in the mitochondrial genome that affect oxidative phosphorylation and mitochondrial proliferation, increased frequency of the common mtDNA deletion in depressed persons, and lower levels of mtDNA expression in depression [95,96,97,98]. Inherited mitochondrial disorders are associated with an increased risk of depression in both pediatric patients and adults [99], where MDD may be the initial symptom of a mitochondrial disorder; depression may affect up to 54% of patients with such disorders [100]. Children with mitochondrial disorders exhibit increased rates of depression compared to the general population [101], with higher rates of mood and anxiety disorders in matrilineal relatives (mitochondrial genes follow a matrilineal pattern of inheritance) [102]. There is also peripheral evidence of abnormal mitochondrial activity in depressed subjects. Gardner et al. [103] performed muscle biopsies on 28 patients with MDD and examined rates of mitochondrial ATP production and enzyme levels, as well as the frequency of mitochondrial genome deletions. They found that, compared to controls, ATP production rates and mitochondrial enzyme levels were lower, and the frequency of mitochondrial genome deletions higher, in the depressed patients. These differences may be due to alterations in mitochondrial genetics.

Biochemical studies of persons with depression indicate altered bioenergetic signatures. Agren and Niklasson demonstrated increased creatine levels in the CSF of persons with MDD, which were positively correlated with CSF levels of dopamine and serotonin metabolites [104]. The authors reported a similar finding in an earlier, smaller study [105]. It has also been shown that peripheral creatine kinase levels are significantly higher in persons with non-psychotic major depression than in other groups of psychiatric patients with psychotic disorders [106].

**Figure 1:**
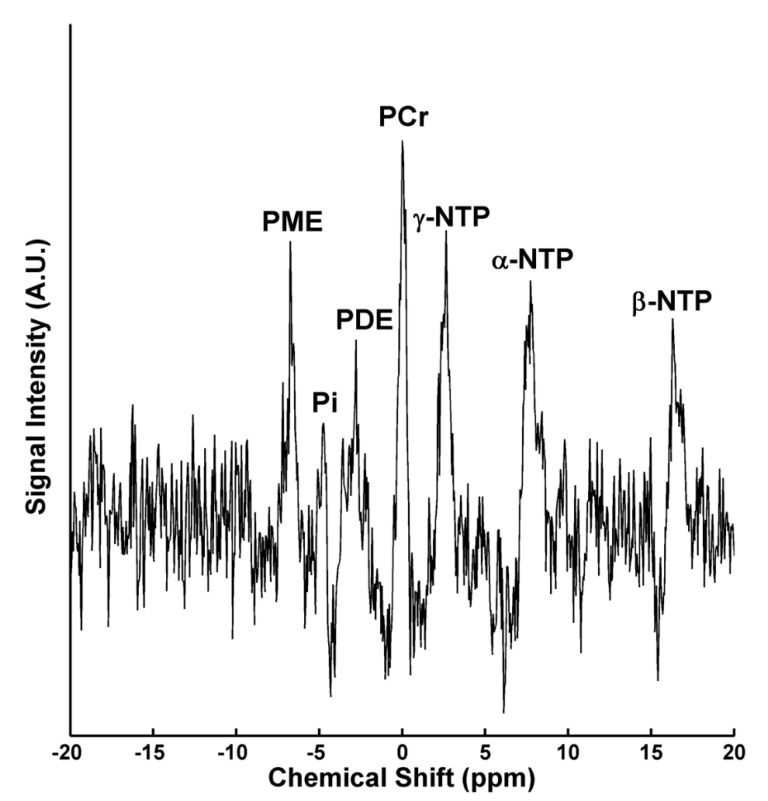
Example phosphorus 31 magnetic resonance spectrum from the frontal lobes of a single subject. Abbreviations: PCr: phosphocreatine; α/β/γ-NTP: α/β/γ-nucleoside triphosphate; PME: phosphomonoester; PDE: phosphodiester; Pi: inorganic phosphate.

Neuroimaging studies also indicate altered bioenergetics in depression. Cerebral glucose metabolism is frequently noted to be abnormal in persons with depression in positron emission tomography studies, particularly in the prefrontal cortex [107,108,109,110,111,112]. Proton (^1^H) and phosphorus (^31^P) magnetic resonance spectroscopy (MRS) studies that allow the measurement of cerebral metabolite concentrations indicate that both unipolar and bipolar depression are associated with alterations in metabolites related to cerebral energy utilization and storage (Figure 1). Although essentially all spectroscopic studies provide at least indirect information about brain energy homeostasis, as they report metabolite levels that indicate the rates of synthetic processes, synaptogenesis, or membrane turnover, we focus here on studies that report directly on the measurement of nucleotide triphosphate (NTP) (which include levels of adenosine triphosphate, the primary energetic compound in cells) and creatine or phosphocreatine (Table 1).

Although several studies in persons with depression have reported no difference in total creatine concentrations ([tCr]), which include concentrations of both creatine ([Cr]) and phosphocreatine ([PCr]), in multiple brain regions [113,114,115,116,117,118,119,120,121,122], others indicate increased [tCr] in some regions, such as in the inferior prefrontal white matter (WM) [123] and left caudate [124]. Studies have shown reduced [tCr] in MDD in the left dorsolateral prefrontal cortex (PFC) [125], posterior cingulate cortex (PCC) [126], and left hippocampus (HC) [127]. A study in of geriatric depression found reduced [tCr] in the PFC in persons with remitted depression compared to healthy controls [128]. It was also found that after electroconvulsive therapy patients with MDD exhibited increases in [tCr] in the dorsal anterior cingulate cortex (ACC) and subgenual ACC.

^31^P MRS studies can measure [PCr] as well as total nucleotide triphosphates ([tNTP]), and beta nucleotide triphosphates ([β-NTP]) such as adenosine triphosphate ([ATP]). Kato et al. (1992) found that [PCr] were significantly reduced in persons with depression compared to persons who were euthymic, with lower [PCr] in those with more severe depression. The study included persons with both BD and MDD [129]. Moore et al. first demonstrated that basal ganglia [β-NTP] were reduced in depressed subjects [130]. Later, it was shown that frontal cortical [β -NTP] were reduced in depressed subject [131]. Renshaw et al. [132] found that, although basal ganglia [β-NTP] and total purine levels did not differ between depressed subjects and healthy controls overall in their sample, in the subgroup of depressed subjects who responded to fluoxetine, [β-NTP] were 21% lower. In female adolescents with depression, baseline depression severity is negatively correlated with [β-NTP] [133]. Volz et al. (1998) found, in subjects with depression who were mostly taking antidepressants, that frontal cortical [ATP] were reduced in depression [131].

In some studies, [PCr] and [B-NTP] have been found to be unchanged in MDD [129,134,135]. One reason for these negative findings, however, may be a failure to segment the brain regions studied into gray matter (GM) and white matter (WM). When segmentation is used, differences in brain energy storage that are intrinsic to the different metabolic properties of GM and WM may be revealed. When patients with MDD were compared to healthy controls and whole brain metabolites were segmented into GM and WM, it was observed that total tissue (GM+WM) [β-NTP] and [tNTP] were lower in depressed subjects and that [tNTP] decreased after 12 weeks of treatment with sertraline [136]. When the authors compared GM and WM, however, they found that [tNTP] was reduced in WM but not in GM before treatment. In a study of older subjects with MDD, again with tissue segmentation, increased WM [β-NTP] and increased GM [PCr] were positively associated with executive function [137]. In a larger study encompassing 50 subjects with MDD, [PCr] was significantly elevated in GM in depression but reduced in WM, while depression ratings were correlated with GM [PCr], but not with WM [PCr] [138].

These findings may indicate that increased [PCr] is associated with depression but is also a marker of antidepressant response-readiness, implying that efforts to increase [PCr] could increase the likelihood of antidepressant response in some patients. This is consistent with a study by Iosifescu et al. [134], which found that subjects who responded to triiodothyronine (T3) exhibited increased [tNTP] but reduced [PCr], while elevated baseline [PCr] predicted response. There are also several reports that [PCr] can increase with antidepressant treatment. Treatment with acetyl-L-carnitine was associated with an antidepressant response and [PCr] in the prefrontal cortex (PFC) increased in tandem with improvements in depression severity [139]. Similarly, adolescent females treated with fluoxetine and adjunctive creatine exhibited increases in [PCr] [133].

Spectroscopic evidence for altered bioenergetics in BD has previously been reviewed [140]. BD has been associated with changes in [tCr]. It appears that [tCr] is reduced in several brain regions in BD, including in the frontal lobes [141], cerebellar vermis [142], hippocampi [143], caudate [144], medial PFC [145,146], dorsolateral PFC WM [146], and PCC [126], although some studies indicate that [tCr] is increased in BD in several brain regions [147,148,149]. Several studies have also failed to find alterations in [tCr], including in the left dorsolateral PFC [122,150,151] and ACC [152,153]. One study found that there were no significant differences in [tCr] between unmedicated BD subjects and controls in a variety of GM and WM regions including the medial frontal cortex, ACC, putamen, caudate, insula, thalamus, parietal cortex, and occipital cortex [147]. The study did, however, find that the severity of bipolar depression was inversely correlated with [tCr].

Using ^31^P MRS, Kato et al. [129] found that [PCr] trended toward being reduced in euthymic subjects with a history of BD. They later demonstrated lower [PCr] in subjects with BD type II compared to controls, though no difference in [PCr] in subjects with BD type I [154]. In a related study, left ventrolateral PFC [PCr] was reduced in euthymic BD subjects compared to controls [155]. It has also been shown that [PCr] is reduced in the whole brain as well as right hemisphere GM in bipolar subjects irrespective of mood state [156]. Other studies indicate that there are no significant differences between bipolar subjects’ [PCr] and those of healthy controls [157,158,159,160,161,162,163,164]. A major limitation of these studies, however, is that subjects were often in different mood states, and bioenergetics markers could vary significantly between mania, euthymia, and depression.

Several studies have suggested dynamic abnormalities in PCr synthesis in BD. In subjects with BD who were treated with lithium, [PCr] fell after photic stimulation (a method of increasing visual cortex activity) in subjects who did not respond to lithium but remained stable in lithium-responsive subjects and controls [161]. This suggested that subjects with BD have a deficit in PCr synthesis that is ameliorated by lithium. In a similar study [163], however, [PCr] fell in response to photic stimulation in controls, but not in bipolar subjects, even though PCr/ATP ratios were reduced in BD, and [ATP] fell in BD in response to photic stimulation. A study using magnetization transfer to estimate the rate constant for the creatine kinase reaction in BD found that it did not differ significantly between euthymic or depressed bipolar subjects and controls [162]. In contrast, subjects with a first episode of bipolar depression (BD) or mania and psychotic features exhibited a 13% reduction in the rate constant for the creatine kinase reaction [159]. The difference between these studies may be due to the absence of psychosis among the subjects in the first study or, again, to the difference in mood states, as many of the subjects in the second study were manic.

### 2.4. Biochemical Effects of Creatine Supplementation

Evidence that brain creatine and phosphocreatine metabolism are altered in depression has suggested that they have promise as antidepressant treatments. Creatine monohydrate, the most common commercially-available form of creatine, has the ability to alter brain creatine levels. The ingestion of 20 g per day of creatine for a month increased brain creatine levels measured by ^1^H MRS by, on average, 4.7% in gray matter and 11.5% in cerebral white matter, though there was significant inter-subject variability that appeared to be related to both gender and body mass [165]. In a placebo controlled study, Lyoo et al. found that supplementation with 0.3 g/kg/d for one week, followed by 0.03 g/kg/d for one week, increased brain [tCr]/[n-acetyl aspartate] ratios by 8.1%, and brain [tCr]/[choline] ratios by 9.3%, as measured by ^1^H MRS. Similar changes were not observed in a placebo control group. The researchers also found, using ^31^P MRS, that creatine supplementation reduced [β-NTP] significantly and produced a trend toward increased [PCr] [166]. The overall effect of creatine supplementation on [PCr] is unclear, but in a study of creatine supplementation in a variety of tissues in several animal species, it appeared that, while the ratio of [PCr] to [Cr] did not change, total [PCr] and [Cr] both increased [167]. This appears to be consistent with findings in human skeletal muscle [168,169]. It was also shown that creatine supplementation at 20g/day x 5 days followed by 5 g/day × 2 days in healthy subjects reduced the fMRI BOLD signal in the V1 region in a visual stimulation paradigm compared to placebo [170]. Creatine supplementation also significantly improved performance on the backward digit span, a test of cognition. The authors speculated that creatine reduces the BOLD signal by increasing local energy stores and thereby reducing the metabolic stimulus for cortical blood flow, or, instead, promoting an increase in the efficiency of O_2_ uptake with associated attenuation of the BOLD signal.

### 2.5. Clinical Studies in Conditions Related to Depression

In human trials, creatine has been studied extensively for the treatment of neuropsychiatric conditions other than depression—especially neurodegenerative illnesses such as Parkinson disease (PD) and Huntington disease (HD), as well as in other neuromuscular disorders. The results of many of these studies are reviewed elsewhere in this issue, but we touch on some of this work here because it provided preliminary clinical evidence that creatine supplementation could improve mood. A 2-year study of the effect of creatine supplementation on the progression of Parkinson disease randomized 60 subjects between placebo or three stages of creatine dosing: 20 g/day for 6 days, 2 g/day for 6 months, and 4 g/day for 18 months. Although there was no significant effect on primary PD symptoms, the investigators observed a significant improvement, relative to placebo, in the “mentation, behavior, mood” subscale of the Unified Parkinson Disease Rating Scale (UPDRS) [171]. Unfortunately, a subsequent multisite study involving 1741 subjects randomized between creatine monohydrate 10 g/day and placebo for 5 years found no difference between groups on the UPDRS mental subscale, nor on the Beck Depression Inventory [172].

A separate study also failed to find evidence of a beneficial effect of creatine on mood in a trial of creatine and strength training in older women (who were without clinical depression at baseline). The women were between the ages of 60 and 80, and were randomized between four arms: creatine alone, placebo alone, creatine plus strength training, and placebo plus strength training. The investigators found that mood changes in the creatine groups did not differ from those in the placebo groups, but that strength training, whether added to creatine or placebo, was associated with improvement in mood.

Persons with depression may exhibit mental fatigue and there are phenomenological similarities between mental fatigue and depression [173,174]. Kato et al. found, in healthy subjects, that the rate of occipital cortex phosphocreatine depletion in response to photic stimulation is associated with the rate of improvement after rest during the Uchida-Kraepelin test (UKT), a paradigm for measuring mental fatigue [175]. In a follow-up to this study, Watanabe et al. discovered that supplementation with 8 g of creatine per day for 5 days reduced mental fatigue on the UKT compared to placebo [176]. Creatine has also been shown to improve cognitive performance in persons subjected to sleep deprivation. Subjects who received 20 g of creatine per day exhibited significantly less decline in cognitive performance, motor performance and mood state than subjects who had received placebo after 24 h of sleep deprivation [177]; in a subsequent study, creatine supplementation improved performance on central executive tasks after 36h of sleep deprivation, though it did not affect mood [178]. Another small study of healthy young adults who were not sleep-deprived found no effect of creatine on cognitive performance compared to placebo over 6 weeks [179]. In the elderly, however, creatine supplementation at 20 g per day for two weeks appears to improve a broad array of cognitive measures [180].

Inspired by findings like those above, Kaptsan and colleagues [181] examined whether creatine could improve neurocognitive and other symptoms in schizophrenia. The investigators randomized 12 patients with schizophrenia to creatine 3 g or 5 g per day or placebo for 3 months in a double-blind, crossover fashion. They found that there was no significant difference between the groups with respect to improvements in neurocognitive function or on study measures such as the Positive and Negative Symptoms Scale (PANSS) or the Clinical Global Impression-Improvement scale, though there were no significant adverse effects. The study did not specifically assay for improvements in mood related to creatine, though no significant difference were observed in the PANSS Negative Symptom or PANSS General Psychopathology subscales, which might indirectly capture depressive symptoms.

Amital et al. (2006) provided oral creatine to subjects with PTSD who were taking SSRIs or SNRIs, with or without comorbid depression. Subjects received 3 g/day of creatine for 1 week, then 5 gm/day for three weeks. The authors found that subjects exhibited significant improvements in the HAM-D, HAM-A, Sheehan Disability Scale, and the Clinician Assessment of PTSD Symptoms, and that improvements were greater in the six subjects who had comorbid MDD [182].

Fibromyalgia is a rheumatological condition that is often associated with depression [183]. Creatine was thought to have potential as a treatment for fibromyalgia because of its benefits for muscle strength and pain [184]. Amital et al. [185] first reported the potential antidepressant benefit of creatine in persons with fibromyalgia based on the experiences of one of the participants in the PTSD study cited above [182]; the patient was a 52-year-old who was treated with creatine in addition to citalopram for 4 weeks. She exhibited improvements in both her Hamilton Depression Rating Scale (HAM-D) scores (which fell from 24 to 16) and symptoms of fibromyalgia. In a randomized, placebo-controlled trial for fibromyalgia lasting 16 weeks, supplementation with creatine at 20 g per day for 5 days, followed by 5 g per day for 15 weeks, produced significant improvements in mental health scores on the Short-Form 36 disability rating scale compared to placebo, though no significant difference was observed in the depression or fatigue subscales of the Fibromyalgia Impact Questionnaire [186].

### 2.6. Clinical Trials of Creatine for Depression

Creatine monohydrate has, to this point, been studied only in small clinical trials for the treatment of MDD, BD, and depression associated with methamphetamine use disorder (see Table 2). With a few exceptions, most trials to date have been positive.

To our knowledge, the first trial to examine creatine for the treatment of depression was conducted by Roitman and colleagues [187]. They examined eight patients with MDD and two patients with BDU and treated them with open-label creatine at 3–5 g/day for four weeks, as an add-on to their existing antidepressants or mood stabilizers. Although both of the patients with BD developed mania/hypomania and were withdrawn from the study, seven out of the eight patients with MDD exhibited significant improvement while receiving creatine. The adverse events reported in the MDD group were mild and transient.

Subsequently, Kondo et al. [133] conducted an open-label trial of creatine supplementation in female adolescents with MDD who had not responded adequately to SSRIs. The study included five girls who had taken fluoxetine for at least 8 weeks but who continued to have clinically significant depressive symptoms, as evidence by a score on the Children’s Depression Rating Scale-Revised (CDRS-R) of at least 40. The subjects were treated with 4g of creatine per day for 8 weeks. The mean CDRS-R score fell by 50.6% between baseline and week 8. The baseline CDRS-R score was correlated with pH as measured by ^31^P MRS, and inversely correlated with [β-NTP]. Creatine-treated subjects exhibited a significant increase in whole brain [PCr] compared to controls.

In a follow up to this study, Kondo and colleagues randomized 34 adolescent and young-adult women with MDD who had not responded to an SSRI to placebo or creatine monohydrate in doses of 2 g, 4 g, or 10 g/day for 8 weeks. They found that creatine increased [PCr] in the frontal cortex compared to placebo, and that higher [PCr] were associated with lower depression scores. There was not a significant difference between creatine groups with respect to the change in [PCr] [170].

In a later study, 52 adult women up to age 65 with depression (HAM-D > 16 and confirmation by the Structured Clinical Interview for DSM-IV) were randomized 1:1 between escitalopram (10 mg per day for one week and then 20 mg per day for seven weeks) plus creatine (3 g per day for one week, then 5 g per day for seven weeks) or matched placebo, to ascertain whether creatine enhanced response to SSRIs. The subjects were otherwise unmedicated before the trial and most (78.8%) were medication-naïve. The creatine-treated group exhibited a superior antidepressant response, compared to the placebo group, as early as week 2, which continued for the 8 weeks of the study; the mean reduction in HAM-D score for the creatine group at week 8 was 79.7%, while in the placebo group it was 62.5%. The creatine group did not experience significantly more adverse effects than the placebo group [188].

Yoon and colleagues later reported neuroimaging results from the women participating in the study above. They hypothesized that creatine administration would increase structural connectivity between rich-club hub network connections, as these connections are energy-demanding. Using ^1^H MRS and structural connectivity imaging, they found that prefrontal N-acetylaspartate levels increased with creatine compared to placebo, and also that rich-club hub network connections in the creatine group increased significantly more than in the placebo group or in healthy controls. There was, however, no evidence that changes in rich-club connectivity were associated with the degree of antidepressant response.

The only negative trial of creatine in MDD to our knowledge was conducted by Nemets and Levine [189]. They enrolled 18 subjects (14 women) who had received an SSRI, SNRI, or noradrenergic and specific serotonergic antidepressant (NaSSA) for at least three weeks and who continued to be depressed in a 4-week trial of adjunctive creatine at 5 g or 10 g per day, or placebo. Although two women receiving in the trial showed an early > 50% reduction in HAM-D scores, overall there was no significant difference between either creatine dose or placebo. The authors concluded that creatine may not be effective for the treatment of depression as an augmenting agent, though it is noteworthy that the period of supplementation in this study was much shorter than in the other, positive, trials.

More recently, our group conducted an open-label trial involving 15 adult women who had failed to respond to adequate trials of at least one SSRI or SNRI, who were treated with 5 g of creatine daily in combination with the serotonin precursor 5-hydroxytryptophan (5-HTP) at 200 mg twice daily for 8 weeks. We found that subjects exhibited significant improvements in HAM-D scores compared to baseline, with an average decrease of approximately 60%. There were no significant adverse events [190].

Although creatine may increase the risk of developing hypomania or mania in persons with bipolar depression [186], it has been investigated in two trials for persons with bipolar depression. In the first trial, Toniolo and colleagues [191] randomized 18 patients with bipolar depression to 6 g of creatine or placebo as augmentation to existing mood-stabilizers or antipsychotics daily for 6 weeks, and assessed the effect on several measures of cognition. They found that subjects taking creatine had a significant improvement in verbal fluency but no significant change in the other measures included. They did not report any effects on the measures of mood included in the study, which included the HAM-D, Montgomery-Asberg Depression Rating Scale (MADRS), and the Young Mania Rating Scale.

In a more recent study, Toniolo and colleagues [192] randomized 53 patients with BD type I or II who were currently in a depressive episode to 6g of creatine daily or matched placebo for 6 weeks, as an adjunct to their existing medications. The researchers did not identify any significant difference between creatine and placebo on the primary endpoint, change in the MADRS after 6 weeks. Strikingly, however, they did find a significant difference in the likelihood of MADRS remission (score <= 12) between the two groups; using intention-to-treat analysis, they found 52.9% remission in the creatine group, with only 11.1% remission in the placebo group. Two patients in the creatine group switched to mania/hypomania early in the study, but no other significant adverse effects were observed.

Hellem et al. (2015) studied the effects of supplementation of 5 g of creatine per day used as monotherapy (i.e., without concomitant antidepressants) on depressive symptoms over 8 weeks in persons with methamphetamine dependence. In this open-label trial involving 14 subjects, the authors found that HAM-D scores were significantly reduced by as soon as 2 weeks after the start of creatine supplementation. They also found that Beck Anxiety Inventory scores were significantly reduced, and that brain [PCr], measured by ^31^P MRS, were significantly increased after 8 weeks. Brain [PCr] were higher at the second ^31^P MRS scan compared to baseline, suggesting that creatine supplementation increased [PCr] [193].

## 3. Conclusions

Creatine is a naturally-occurring organic acid that serves as an energy buffer and energy shuttle in tissues, such as brain and skeletal muscle, that exhibit dynamic energy requirements. Evidence, deriving from a variety of scientific domains, that brain bioenergetics are altered in depression and related disorders is growing. Clinical studies in neurological conditions such as PD have indicated that creatine might have an antidepressant effect, and early clinical studies in depressive disorders—especially MDD—indicate that creatine may have an important antidepressant effect. Future work should, we think, involve larger clinical trials of creatine when used as an adjunctive treatment in MDD, extend to encompass trials of creatine as monotherapy, examine the potential efficacy of creatine as an augmenting agent when combined with neurostimulation techniques such as ECT and TMS, and better characterize the neurochemical and network-level effects of creatine and their correlations with antidepressant response. 

## Figures and Tables

**Table 1:** Studies reporting phosphocreatine and total creatine levels in major depressive disorder.

Study	Condition/Control	Brain Region	Change Compared to Controls
*^31^P-MRS Studies reporting phosphocreatine levels in MDD and Bipolar Disorder*			
Kato 1992 [129]	MDD-D/MDD-E	30mm frontal axial slice	None
Kato 1994 [154]	BDII/HC	30mm frontal axial slice	↓
	BDI/HC	30mm F axial slice	None
Murashita 2000 [161]	BD/HC (+ PS)	OCC	↓ after PS in BD except in lithium responders
Pettegrew 2002 [139]	MDD/HC	PFC	↑ after treatment associated with AD response
Iosifescu 2008 [134]	MDD/HC	20 mm-thick axial slice	None overall but ↓ baseline PCr in those who responded to T3
Sikoglu 2013 [164]	BD/HC	FL	None
Weber 2013 [155]	BD-E/HC	L VLP FC	↓
Yuksel 2015 [163]	BD/HC (+ PS)	OCC	↓ after PS in HC but not BD; no difference in PCr at baseline
Dudley 2016 [156]	BD/HC	WB WM	↓
Harper 2016 [137]	MDD/HC	WB WM, WB GM	↑
Harper 2017 [138]	MDD/HC	WB WM, WB GM	↑ in GM, ↓ in WM
*^1^H-MRS Studies Reporting total creatine levels in MDD and Bipolar Disorder*			
Hamakawa 1999 [141]	BD-D/BD-E	L FL	↓
Auer 2000 [113]	MDD/HC	B A CC	None
Farchione 2000 [114]	MDD/HC	B DL PFC	None
Kumar 2002 [115]	MDD/HC	DL WM, ACC	None
Cecil 2003 [142]	BD-D/HC	CV	↓
Deicken 2003 [143]	BD- E/HC	B Hippo	↓
Gruber 2003 [123]	MDD/HC	L PF WM	↑
Michael 2003 [122]	MDD/HC	L AMG	None
Pfleiderer 2003 [117]	MDD/HC	L A CC	None
Dager 2004 [147]	BD/HC	FL WM, BG, Thal	tCr inversely correlated with depression severity
Brambilla 2005 [150]	BD/HC	L DL PFC	None
Mirza 2006 [120]	MDD/HC	B Thal	None
Frye 2007 [150]	BD-D/HC	A CC, M CC, M PFC	↑
Gabbay 2007 [124]	MDD/HC	L CN	↑
Moore 2007 [152]	BD/HC	B ACC	None
Olvera 2007 [151]	BD/HC	L DLPFC	None
Patel 2008 [149]	BD-D/HC	B VL PFC	↑
Port 2008 [144]	BD-D/HC	R CN	↓
Nery 2009 [125]	MDD/HC	L DL PFC	↑ in women ↓ in men
Öngür 2009 [153]	BD-M/HC	B ACC and POC	None
Venkatraman 2009 [128]	MDD/HC	M PFC	↓
Caetano 2011 [147]	BD/HC	R M PFC, L DL PFC WM	↓
Portella 2011 [118]	MDD/HC	B VM PFC	None
McEwen 2012 [116]	PPD/HC	B M PFC	None
Özdel 2012 [145]	BD-E/HC	B M PFC	↓
Bradley 2016 [121]	MDD/HC	B CN, B Put, B Thal	None
Li 2016 [126]	MDD/HC	P CC	↓
Njau 2017 [127]	MDD/HC	SG ACC, D ACC	↑
Rosa 2017 [119]	PPD/HC	B A CC, L DL PFC	None

A: anterior; AMG: amygdala; B: bilateral; BD: bipolar disorder; BD-D: bipolar, depressed state; BD-E: bipolar, euthymic state; BDI: bipolar disorder type I; BDII: bipolar disorder type II; BD-M: bipolar, manic or mixed state; BG: basal ganglia; CC: cingulate cortex; CV: cerebellar vermis; D: dorsal; DL: dorsolateral; FL: frontal lobes; GM: gray matter; HC: healthy controls; Hippo: hippocampus; Ins: insula; L: left; M: medial; MCC: middle cingulate cortex; MDE: major depressive episode; MDD: major depressive disorder; MDD-D: major depressive disorder, depressed state; MDD-E; major depressive disorder, euthymic state; M: medial; OCC: occipital cortex; OFC: orbitofrontal cortex; OL: occipital lobe; P: posterior; PCr: phosphocreatine; PFC: prefrontal cortex; POC: parieto-occipital cortex; PPD: post-partum depression; PS: photic stimulation; Put: putamen; R: right; SG: subgenual; tCr: total creatine (phosphocreatine + creatine); Thal: thalamus; TL: temporal lobes; T3: triiodothyronine; V: ventral; VL: ventrolateral; WB: whole brain; WM: white matter; ↑: significantly increased/higher; ↓: significantly reduced/lower.

**Table 2:** Clinical trials involving creatine for the treatment of depression.

Study	Population (*n*)	Design	Creatine Dose	Duration	Effect	Significant Adverse Effects Related to Creatine
Roitman 2007 [186]	MDD-D (*n* = 8); BD-D (*n* = 2)	Open-label, adjunctive	3–5 g/day	4 weeks	Average HAM-D scores declined from 23.1 at baseline to 12.6 at week 4	Both bipolar subjects developed hypomania/mania
Kondo 2011 [133]	Adolescent girls with MDD-D (*n* = 5)	Open-label, adjunctive	4 g/day	8 weeks	The mean CDRS-R score fell by 50.6%	None
Kondo 2016 [170]	Adolescent and young-adult women with MDD-D (*n* = 34)	Open-label, adjunctive, dose-ranging	2 g, 4 g, or 10 g/day	8 weeks	Creatine increased frontal cortical phosphocreatine levels in a fashion associated with lower depression ratings	None
Lyoo 2012 [188]	Women with MDD-D (*n* = 52)	Randomized, double-blind, placebo-controlled, adjunctive	3 g/day × 1 week then 5 g/day × 7 weeks	8 weeks	HAM-D scores in the creatine group fell by 79.7% by week 8, compared to 62.5% in the placebo group	None
Nemets 2013 [189]	MDD-D (*n* = 18)	Randomized, double-blind, placebo-controlled, adjunctive	5 g/day or 10 g/day	4 weeks	No significant difference between creatine and placebo in HAM-D scores	None
Hellem 2015 [193]	Methamphetamine dependence with depression (*n* = 14)	Open-label, monotherapy	5 g/day	8 weeks	Mean HAM-D scores fell to 10.4 by week 2, representing response	Gastrointestinal symptoms (*n* = 5) and muscle cramps (n = 2)
Kious 2017 [190]	Women with MDD-D (*n* = 15)	Open-label, adjunctive	5 g/day (with 5-HTP 200 mg twice daily)	8 weeks	HAM-D scores improved by ~60% by week 8	None
Toniolo 2017 [191]	BD-D (*n* = 18)	Randomized, double-blind, placebo-controlled, adjunctive	6 g/day	6 weeks	Significant improvement in verbal fluency but no significant changes in other measures reported	None
Toniolo 2018 [192]	BD-D (*n* = 53)	Randomized, double-blind, placebo-controlled, adjunctive	6 g/day	6 weeks	No significant difference in MADRS scores between groups, but MADRS remission rate was significantly greater in creatine group (52.9% vs. 11.1%)	Two participants in creatine group developed hypomania/mania

BD-D: bipolar disorder, depressed state; CDRS-R: Children’s Depression Rating Scale, Revised; HAM-D; 17-item Hamilton Depression Rating Scale; MADRS: Montgomery-Asberg Depression Rating Scale MDD-D: major depressive disorder, depressed state.
